# Association of SCN1A, SCN2A, and UGT2B7 Polymorphisms with Responsiveness to Valproic Acid in the Treatment of Epilepsy

**DOI:** 10.1155/2020/8096235

**Published:** 2020-02-25

**Authors:** Yuan Lu, Quanping Su, Ming Li, Alimu Dayimu, Xiaoyu Dai, Zhiheng Wang, Fengyuan Che, Fuzhong Xue

**Affiliations:** ^1^Department of Biostatistics, School of Public Health, Shandong University, Jinan, 250012 Shandong, China; ^2^Healthcare Big Data Institute of Shandong University, Jinan, 250012 Shandong, China; ^3^Central Laboratory, Linyi People's Hospital, Linyi, 276003 Shandong, China; ^4^Linyi People's Hospital, Linyi, 276003 Shandong, China; ^5^Department of Neurology, Linyi People's Hospital, Linyi, 276003 Shandong, China

## Abstract

**Purpose:**

The efficacy of valproic acid (VPA) varies widely in clinical treatment of epileptic patients. Our study is aimed at exploring a potential association between polymorphisms of SCN1A, SCN2A, and UGT2B7 genetic factors and VPA responses.

**Methods:**

In this observational study, a total of 114 epileptic patients only treated with VPA for at least 1 year were included to explore the genetic polymorphisms of drug responses (mean follow-up time: 3.68 ± 1.78 years). Thirty-one single-nucleotide polymorphisms (SNPs) in three candidate genes that related with drug-metabolizing enzymes and receptors were genotyped.

**Results:**

Of the 31 SNPs, eight were significantly associated with VPA responses, including rs1381105, rs2162600, rs10197716, rs2119068, rs2119067, rs353116, rs353112 and rs6740895. The interaction between rs10197716 and rs2119068 was the most significantly correlated with VPA responses compared with other combinations (the highest VPA-responsive rate 0.92 versus the lowest VPA-responsive rate 0.33, *p* = 0.007).

**Conclusion:**

The study indicated that eight SNPs and SNP-SNP interaction may be associated with VPA responses in Chinese Han epileptic patients. The SNPs were rs1381105 (SCN1A), rs2162600 (SCN1A), rs10197716 (SCN2A), rs2119068 (SCN2A), rs2119067 (SCN2A), rs353116 (SCN2A), rs353112 (SCN2A) and rs6740895 (SCN2A), respectively. The interaction between the three pairs of rs10197716-rs2119068, rs10197716-rs11889342 and rs7598931-rs12233719 was the most significant for VPA. This implied that these SNPs may play an important role in the pharmacogenomics mechanism of valproic acid.

## 1. Introduction

Epilepsy is a chronic neurological disease of brain dysfunction that has received widespread attention in society [1, 2]. Recent studies have shown that a total of 45.9 million people suffer from epilepsy in the world, which is one of the important causes of disability and death in the population [3].

Traditional antiepileptic drugs include valproic acid, carbamazepine and phenytoin. Valproic acid is a commonly used broad-spectrum antiepileptic drug, which is effective for various types of seizures, including tonic-clonic, myoclonic, and absence seizures [4]; carbamazepine is also commonly used as first-line antiepileptic drugs for focal onset seizures but may not be effective for absence or myoclonic seizures; phenytoin is mainly used to treat partial seizures, but no longer considered a first-line treatment due to concerns over adverse events [5]. After taking the above drugs, 20%-40% of patients with epilepsy still cannot effectively control seizures [6]. The mechanism of drug resistance has not been fully explained, but genetic factors have been recognized as an important cause of drug efficacy difference in the treatment of epilepsy [7, 8]. Drug-metabolizing enzymes, transporter, and receptor variants may be due to genetic polymorphisms that affect clinical outcomes in different AED therapy, and these clinical implications of gene polymorphisms associated with antiepileptic drugs have been reported [9]. In terms of drug metabolism enzyme genes, the CYP2C9 polymorphisms were associated with the dosage of phenytoin, and the widely studied CYP2C19 showed that different haplotypes had different toxicities, when epileptic patients took valproic acid [10, 11]. Polymorphisms of UGT2B7 affected the metabolism of AEDs such as VPA, CBZ and lamotrigine (LTG), and UGT2B7 was associated with oxcarbazepine (OXC) maintenance doses, which were useful for the personalization of OXC therapy [12, 13]. Some studies on ABCB1, a drug transporter gene, indicated that genetic polymorphisms were associated with resistance of AEDs in patients with epilepsy [14, 15]. SCN1A and SCN2A genes encoding the sodium channels were related to the efficacy, dose, and toxicity of multiple antiepileptic drugs [16]. These two genes were reported to be associated with efficacy of mono- or combination antiepileptic therapy, but the results were contradicted in different studies. For example, SCN1A (rs2298771) was correlated with responses to AEDs in epileptic patients [17], while some studies found that the SNP had no association with drug responses, similar in SCN2A [18]. As a key metabolic enzyme gene, UGT2B7 plays a crucial role in the metabolism of valproic acid; SCN1A and SCN2A are key genes encoding sodium channels. These gene mutations affect drug responsiveness in epilepsy. At present, the research results on the relationship between the above three genes and various antiepileptic drugs are not consistent. And current researches could not include all SNPs on these three genes. Therefore, based on the study of Han population taking valproic acid, new SNPs located in the SCN1A, SCN2A, and UGT2B7 were studied.

In summary, although the mechanism of AED responses has been still unclear, many studies have shown that the individual genetic variations were related to the responses of antiepileptic drugs. It is known that VPA is a broad-spectrum antiepileptic drug that is commonly used to control seizures [19]. And SCN1A, SCN2A, and UGT2B7 were reported that they are related to the efficacy of VPA by encoding sodium voltage-gated channels or influencing uridine 5′-diphospho-glucuronosyltransferases (UGTs) [20, 21]. In order to explore the associations between common SCN1A, SCN2A, and UGT2B7 gene polymorphisms, alone or in combination, with VPA responsiveness in epilepsy among 114 Chinese Han patients, we carried out a pharmacogenomics association analysis to identify whether genetic polymorphisms were correlated with VPA responses using a total of 31 SNPs in the three candidate genes.

## 2. Methods

### 2.1. Participants

Participants of this study were derived from the demonstration platform of the epilepsy follow-up cohort of Linyi People's Hospital that constructed a long-term follow-up cohort. This platform was based on the clinical diagnosis and treatment records of patients with epilepsy. Baseline information, medical information, and frequency of epileptic seizures after the patient entered the cohort were recorded. We screened participants who had been followed up for more than one year and had been taking valproic acid. Finally, the study included 114 epileptic patients who received treatment with valproic acid from Linyi City People's Hospital. The research was approved by the ethics committee of School of Public Health, Shandong University in China. All participants or their legal guardian signed the written informed consent. All epileptic patients were diagnosed by clinicians according to the criteria of International League Against Epilepsy in combination with clinical manifestations and electroencephalograms [22]. Exclusion criteria included the presence of mental illness, such as autism, schizophrenia and affective disorder, presence of progressive or degenerative neurological disease, presence of any malignant tumor disease or severe organic heart disease or liver and kidney disease, history of alcohol or drug abuse and incomplete record of seizure frequency. For each participant, basic information was collected: age at first visit, gender, epilepsy etiology, seizure frequency and VPA dosage (mg). Patients were defined as VPA-responsive epilepsy if they have not experienced any type of seizure for at least 12 months after treatment with VPA. The remaining patients were included as VPA-resistant epilepsy [23].

### 2.2. Genotyping

Peripheral venous blood samples (5 ml) were collected from each subject in Eppendorf tubes containing ethylenediaminetetraacetic acid (EDTA). AssayDesigner3.1 software (Sequenom, Inc., CA, USA) was used for primer design and multiplex reactions according to actual conditions. The studied genetic polymorphisms were genotyped by using Sequenom MassARRAY System and MALDI-TOF mass spectrometry. Based on previous studies, we finally selected SCN1A, SCN2A and UGT2B7 [24–27]. The following criteria were used to select SNPs from the above genes [28]: selected SNPs has been reported to be related to nervous system diseases in previous studies; partially representative SNPs have been selected for too many SNP locus and high linkage disequilibrium (LD) *r*^2^ > 0.8 in block; for some genes with limited coding SNPs, some noncoding SNPs were also included according to LD.

### 2.3. Statistical Analysis

Minor allele frequency (MAF), Hardy–Weinberg equilibrium (HWE) test and linkage disequilibrium (LD) were conducted using Haploview 4.2 software (https://www.broadinstitute.org/haploview/haploview). We choose the default “confidence intervals (Gabriel et al.)” to define blocks [29]. And R software (version 3.5.1 for Windows, https://www.r-project.org/) was used to perform univariate analysis, logistic regression analysis and SNP-SNP interaction analysis. Basic characteristics including continuous and categorical variables were described using Student's *t*-test or chi-square test (Fisher's exact test). The association analysis between genotypes or alleles and drug response was tested using binary logistic regression with adjusting other covariates, including age, gender and VPA dosage. The criteria to select candidate SNPs were given as follows: MAF > 0.05, HWE > 0.05, and detection rate of each SNPs > 95%. *p* value < 0.05 is considered statistically significant.

## 3. Result

The sample population consisted of 114 patients with epilepsy: 90 VPA responders (mean follow-up time: 3.76 ± 1.77 years) and 24 VPA resisters (mean follow-up time: 3.40 ± 1.84 years). The characteristics including age, gender, VPA dosage and epilepsy etiology were not significant between the VPA-responsive and VPA-resistant groups. The basic characteristics of the study population are described in Table 1.

**Figure 1:**
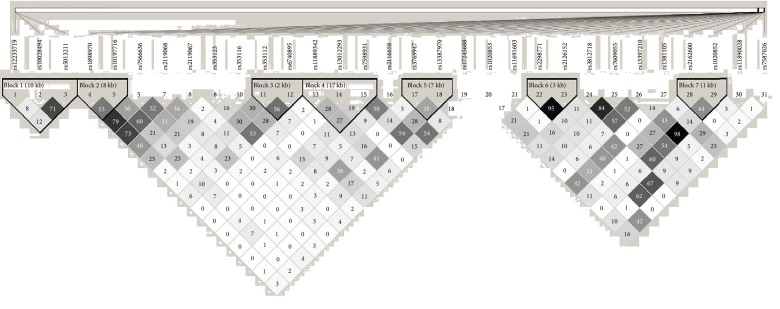
Linkage disequilibrium (LD) of SCN1A, SCN2A, and UGT2B7 for 31 single-nucleotide polymorphisms (SNPs) in 114 patients. Numbers 1-3 were in UGT2B7; 4-19 were in SCN2A; 20-31 were in SCN1A. Haploview analysis of LD (*r*^2^) between pairwise comparisons of SNPs <500 kb apart is shown in the figure, with a darker square for larger *r*^2^. Seven haplotype blocks were identified using confidence intervals algorithm.

All studied SNPs conformed to HWE > 0.05 and MAF > 0.05. Linkage disequilibrium blocks are displayed in Figure 1. The figure showed that SNP-SNP LD information was located in SCN1A, SCN2A and UGT2B7 genes. According to confidence intervals algorithm, seven haplotype blocks were identified. The frequency of a haplotype block did not differ significantly among VPA-responsive and VPA-resistant group.

Among the studied SNPs, there were eight SNPs associated with VPA efficacy in the SCN1A, SCN2A, and UGT2B7, summarized in Table 2. The analysis of SCN1A showed that GT genotype of rs1381105 (0.36 (95% CI: 0.14-0.94)), TC genotype, “TC+CC” combination genotype and allele C of rs2162600 (0.34 (95% CI: 0.13-0.92), 0.36 (95% CI: 0.14-0.92), and 0.46 (95% CI: 0.22-1.02), respectively) were significantly associated with VPA resistance. The rest of the SNPs associated with VPA responses belonged to SCN2A, which were TC genotype of rs2119067 (0.37 (95% CI: 0.13-0.96)), CT, CC genotype, “CT+CC” combination genotype and allele C of rs353116 (2.81 (95% CI: 1.01-8.43), 5.83 (95% CI: 1.24-45.05), 2.34 (95% CI: 1.27-9.37) and 2.62 (95% CI: 1.27-5.80), respectively), GG genotype and allele G of rs10197716 (0.19 (95% CI: 0.04-0.78) and 0.46 (95% CI: 0.23-0.90), respectively), GG genotype, “CG+GG” combination genotype and allele G of rs2119068 (9.09 (95% CI: 1.51-176.64), 3.01 (95% CI: 1.15-8.03), and 2.48 (95% CI: 1.22-5.33), respectively), “GC+CC” combination genotype and allele C of rs353112 (95% CI: 3.16 (1.21-8.91) and 3.07 (95% CI: 1.39-7.60), respectively), CT genotype, “CT+CC” combination genotype and allele C of rs6740895 (5.80 (95% CI: 1.65-28.94), 7.02 (95% CI: 2.03-34.46) and 5.91 (95% CI: 1.96-25.84), respectively). Therefore, genetic variation might be considered a potential predict factor for drug resistance.

**Figure 2:**
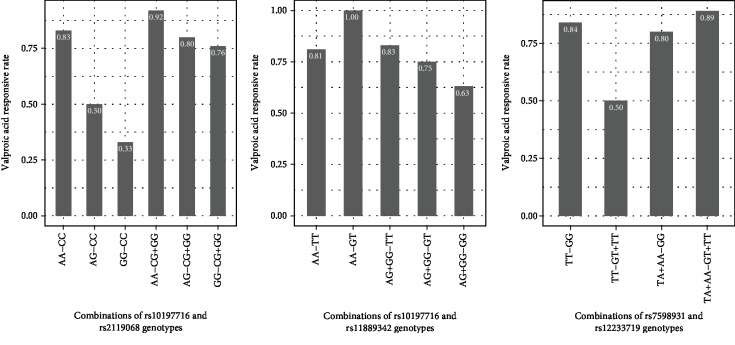
VPA-responsive rate among different combinations of genotypes. The responsive rate of AA-CG+GG group or AG-CG+GG group was significantly higher than that of the GG-CC group (*p* values were 0.007 and 0.028, respectively) in (a). The responsive rate of AG+GG-GT group or AG+GG-GG group was significantly lower than that of the AA-GT group (*p* values were 0.047 and 0.013, respectively) in (b). The responsive rate of TA+AA-GG group, TT-GG group, or TA+AA-GT+TT was significantly higher than that of the TT-GT+TT group (*p* values were 0.046, 0.016, and 0.023, respectively) in (c). VPA: valproic acid.

The significant association between SNP interactions and VPA responses is summarized in Table 3. A total of 19 pairs of SNP-SNP interactions were related to the drug efficacy based on “codominant (epistasis)” model. VPA-responsive rates with different genotype combinations of three pairs (*p* < 0.01) are descripted in Figure 2. They were rs10197716 (SCN2A)-rs2119068 (SCN2A), rs10197716 (SCN2A)-rs11889342 (SCN2A), and rs7598931 (SCN2A)-rs12233719 (UGT2B7), respectively. Since GG carriers were few in rs2119068, genotype CG and GG were merged into one group, namely, the CG+GG group. Similar processes were also conducted in rs10197716, rs7598931, and rs12233719 for the same reason. The VPA-responsive rate of GG-CC carriers was only 0.33, while the carriers of AA-CG+GG (combination of AA in rs10197716 and CG+GG in rs2119068) and AG-CG+GG (combination of AG and CG+GG) were higher than that of the GG-CC group (0.92 vs. 0.33 and 0.80 vs. 0.33, *p* < 0.05), which was about 2.5 times that of the GG-CC group in Figure 2(a). The AG+GG-GT (combination of AG+GG in rs10197716 and GT in rs11889342) group and AG+GG-GG (combination of AG+GG and GG) group, 0.75 and 0.63, respectively, were lower than that the AA-GT group (*p* < 0.05), which was nearly 1.00 with VPA-responsive rate in the combinations of rs10197716 and rs11889342 genotypes shown in Figure 2(b). The TT-GT+TT (combination of TT and GT+TT) group was significantly lower than the other three combinations (*p* < 0.05) from rs7598931 and rs12233719 showed in Figure 2(c). It is worth noting that the interaction of rs7598931 and rs12233719 (*p* < 0.001), which were in SCN2A and UGT2B7 respectively, were associated with the VPA responsiveness, but these two SNPs were not associated with VPA responses in the univariate logistic analysis. Responsive rates of other combinations (SNP-SNP *p* value > 0.01 and *p* < 0.05) need to be explored further.

## 4. Discussion

In our study, we found the relationship between the SNPs screened from SCN1A, SCN2A and UGT2B7, respectively, and VPA responses in Chinese Han people with epilepsy. Our results indicated that two SNPs (rs1381105 and rs2162600) located in SCN1A and six SNPs including rs10197716, rs2119068, rs2119067, rs353116, rs353112 and rs6740895 located in SCN2A had different distributions in the VPA resistance group and the responsive group, which were possible for altering sensitivity for VPA. Through SNP-SNP interaction analysis, a total of 19 pairs of SNPs were associated with VPA responsiveness. The three most obvious pairs were rs10197716 (SCN2A)-rs2119068 (SCN2A), rs10197716 (SCN2A)-rs11889342 (SCN2A) and rs7598931 (SCN2A)-rs12233719 (UGT2B7). In comparison with previous studies, we found that some of our findings were controversial or novel.

The pathogenesis of epilepsy is complex and is thought to be due to excitatory and inhibitory imbalances in the central nervous system currently, which is closely related to neurotransmitter imbalance and ion channels [30]. It has been well documented that changes in expression and affinity of binding site in the sodium channels (SCNs) may be resistant to AEDs [31]. And VPA is a kind of histone deacetylase inhibitor that inhibit the voltage-sensitive sodium channels of neuronal firing to exert neurosuppressive effects [32]. Therefore, SCN gene mutation encoding sodium channels may lead to abnormal receptor function and affect the efficacy of VPA. rs6740895 is an intron variant located in SCN2A that encodes sodium voltage-gated channel alpha subunit 2. In the association analysis, we found that the distribution of genotypes with rs6740895 (SCN2A) was significantly different in VPA resistance group and responsive group, and allele C was associated with VPA responsiveness. Until now, the correlation between this site mutation and the efficacy of antiepileptic drugs has not been reported, and the detailed function of this site has not been explained clearly. The possible mechanism that the SNP alters the VPA response may be to alter the structure or function of the sodium channel receptors [33]. rs2298771 is a kind of missense variant in SCN1A, which causes the change of alanine to threonine [34]. However, we did not find a correlation between the SNP and VPA response. In fact, the result of rs2298771 (SCN1A) associated with response of AEDs so far is controversial. A few studies found that mutant genotype or allele did not improve or decrease efficacy with taking mono- or combination antiepileptic drugs, but some studies indicated that genetic polymorphism had a significant correlation with responsiveness of AEDs [24, 35]. The SNP rs3812718 an intron variant located in SCN1A had also different results in a variety of studies. 5′-diphosphate glucuronosyltransferase is essential for drug metabolism. As an important gene of this enzyme, UGT2B7 plays an important role in VPA metabolism. Wang et al. did not identify the relationship between rs12233719 (UGT2B7) and VPA metabolism in a meta-analysis [13]. Our study found that there was no correlation between this locus and the efficacy of VPA. There are several reasons for inconsistent results in different studies. Firstly, sample size is a key factor in the association analysis, which may cause false positive and low power. Secondly, genetic differences in different races may be also an important factor. Thirdly, different treatments, mono- or combination AEDs, may also affect treatment results of AEDs [17, 36, 37]. A SNP-SNP interaction, rs7598931 and rs12233719 located in SCN2A and UGT2B7 separately, associated with VPA responses was first found. It is amazing that these SNPs were not associated with VPA responses alone in our association analysis. The interaction analysis implied that a potential association between combinations of SNP-SNP genotypes and VPA responses in the association study could be ignored possibly. More interaction analysis may help discover new association between SNP-SNP interaction and drug responsiveness. At present, it is not clear whether the interactions of different SNP pairs are truly associated with VPA responses, and mechanisms remain to be studied. Therefore, the molecular mechanism is required to further research.

We studied the association between genetic polymorphisms and mono antiepileptic therapy (VPA), avoiding confounder for multiple drug interactions or competing for common targets [38]. Meanwhile, multiple SNPs that have never been reported in the past were chosen in Chinese Han epilepsy patients. This not only reveals new SNP locus information but also avoids racial differences. Moreover, we have also discovered the interaction among different SNPs for the first time, while the mechanism will be needed for further exploration. It is worth noting that some limitations existed in our study. Firstly, the sample size was relatively small compared with previous studies, which may reduce the statistical power. Secondly, our research sample lacked some recorded factors that had been reported in previous studies, such as age of onset and seizure type. Thirdly, the studied SNPs do not consist of all genetic information, so other mutations that were not covered in this study may also be associated with VPA responses. Further research based on enough sample size is not only to explore more potential gene mutations but also to clarify mechanisms and pathways with VPA responses.

## 5. Conclusion

Our study indicated that multiple SNPs, including rs1381105 (SCN1A), rs2162600 (SCN1A), rs10197716 (SCN2A), rs2119068 (SCN2A), rs2119067 (SCN2A), rs353116 (SCN2A), rs353112 (SCN2A) and rs6740895 (SCN2A), especially the SNP-SNP interaction (rs10197716-rs2119068, rs10197716-rs11889342 and rs7598931-rs12233719) were associated with VPA responsiveness in Chinese Han patients with epilepsy. Such gene mutations were found to potentially affect drug responses, facilitating personalized treatment for VPA in epileptic patients.

## Figures and Tables

**Table 1:** Basic characteristics of the epileptic patients.

Characteristics	VPA responsive (*n* = 90)	VPA resistant (*n* = 24)	Total (*n* = 114)	*p* value
Age (years)	29.17 ± 14.26	26.29 ± 13.16	28.56 ± 14.02	0.373
Dose (mg)	463.28 ± 288.00	569.58 ± 268.22	485.66 ± 286.12	0.103
Gender				
Male	65 (72.2)	18 (75.0)	83 (72.8)	0.989
Female	25 (27.8)	6 (25.0)	31 (27.2)	
Etiology				
Idiopathic	68 (75.6)	13 (54.2)	81 (71.1)	0.122
Symptomatic	20 (22.2)	10 (41.7)	30 (26.3)	
Cryptogenic	2 (2.2)	1 (4.2)	3 (2.6)	

Data were presented as mean ± standard deviation or *n* (%). VPA: valproic acid.

**Table 2:** Genotypes/alleles frequencies of SCN1A (2A) and UGT2B7 in VPA responsive and VPA resistant groups (*p* < 0.05).

Genes	SNPs	Genotypes/alleles	VPA responsive	VPA resistant	OR (95% CI)	*p* value
SCN1A	rs1381105	GG	57 (63.3)	11 (45.8)	Reference	
		GT	27 (30.0)	13 (54.2)	0.36 (0.14-0.94)	0.039
		TT	6 (6.7)	0 (0.0)	—	
		GT+TT	33 (36.7)	13 (54.2)	0.49 (0.19-1.229)	0.129
		G	141 (78.3)	35 (72.9)	Reference	
		T	39 (21.7)	13 (27.1)	0.78 (0.38-1.69)	0.516

SCN1A	rs2162600	TT	65 (72.2)	12 (50.0)	Reference	
		TC	22 (24.4)	11 (45.8)	0.34 (0.13-0.92)	0.033
		CC	3 (3.3)	1 (4.2)	0.46 (0.05-9.96)	0.524
		TC+CC	25 (27.8)	12 (50.0)	0.36 (0.14-0.92)	0.033
		T	152 (84.4)	35 (72.9)	Reference	
		C	28 (15.6)	13 (27.1)	0.46 (0.22-1.02)	0.049

SCN2A	rs10197716	AA	27 (30.0)	3 (12.5)	Reference	
		AG	45 (50.0)	12 (50.0)	0.35 (0.07-1.31)	0.148
		GG	18 (20.0)	9 (37.5)	0.19 (0.04-0.78)	0.030
		AG+GG	63 (70.0)	21 (87.5)	2.50 (0.89-6.67)	0.075
		A	99 (55.5)	18 (37.5)	Reference	
		G	81 (45.5)	30 (62.5)	0.46 (0.23-0.90)	0.024

SCN2A	rs2119068	CC	24 (26.7)	12 (52.2)	Reference	
		CG	49 (54.4)	10 (43.5)	2.37 (0.88-6.61)	0.091
		GG	17 (18.9)	1 (4.3)	9.09 (1.51-176.64)	0.045
		CG+GG	66 (73.3)	11 (47.8)	3.01 (1.15-8.03)	0.025
		C	97 (53.9)	34 (73.9)	Reference	
		G	83 (46.1)	12 (26.1)	2.48 (1.22-5.33)	0.015

SCN2A	rs2119067	TT	52 (57.8)	10 (41.7)	Reference	
		TC	34 (37.8)	14 (58.3)	0.37 (0.13-0.96)	0.045
		CC	4 (4.4)	0 (0.0)	—	
		TC+CC	38 (42.2)	14 (58.3)	0.40 (0.15-1.05)	0.067
		T	138 (76.7)	34 (70.8)	Reference	
		C	42 (23.3)	14 (29.2)	0.62 (0.30-1.33)	0.209

SCN2A	rs353116	TT	30 (34.5)	14 (58.3)	Reference	
		CT	41 (47.1)	8 (33.3)	2.81 (1.01-8.43)	0.053
		CC	16 (18.4)	2 (8.3)	5.83 (1.24-45.05)	0.046
		CT+CC	57 (65.5)	10 (41.7)	2.34 (1.27-9.37)	0.017
		T	101 (58.0)	36 (75.0)	Reference	
		C	73 (42.0)	12 (25.0)	2.62 (1.27-5.80)	0.012

SCN2A	rs353112	GG	38 (42.2)	16 (66.7)	Reference	
		GC	40 (44.4)	8 (33.3)	2.40 (0.91-6.83)	0.084
		CC	12 (13.3)	0 (0.0)	—	
		GC+CC	52 (57.8)	8 (33.3)	3.16 (1.21-8.91)	0.022
		G	116 (64.4)	40 (83.3)	Reference	
		C	64 (35.6)	8 (16.7)	3.07 (1.39-7.60)	0.009

SCN2A	rs6740895	TT	53 (58.9)	21 (87.5)	Reference	
		CT	30 (33.3)	3 (12.5)	5.80 (1.65-28.94)	0.014
		CC	7 (7.8)	0 (0.0)	—	
		CT+CC	37 (41.1)	3 (12.5)	7.02 (2.03-34.46)	0.006
		T	136 (75.6)	45 (93.8)	Reference	
		C	44 (24.4)	3 (6.2)	5.91 (1.96-25.84)	0.005

OR: odds ratio; CI: confidence interval; SNPs: single-nucleotide polymorphisms; VPA: valproic acid. Due to no significant SNP in the recessive model, it is not listed in the table.

**Table 3:** SNP-SNP interaction analysis among 31 studied single-nucleotide polymorphisms.

Chromosome	Gene	SNPs	Chromosome	Gene	SNPs	*p* value
2:166122240	SCN1A	rs7587026	2:165270773	SCN2A	rs2119067	0.046
2:166035836	SCN1A	rs11691603	2:165270773	SCN2A	rs2119067	0.025
2:166069891	SCN1A	rs2162600	2:165307150	SCN2A	rs7598931	0.033
2:166035836	SCN1A	rs11691603	2:165283510	SCN2A	rs6740895	0.028
2:166122240	SCN1A	rs7587026	2:165283510	SCN2A	rs6740895	0.026
2:165310162	SCN2A	rs2116658	2:165268464	SCN2A	rs10197716	0.029
2:165290104	SCN2A	rs11889342	2:165283510	SCN2A	rs6740895	0.048
2:165337455	SCN2A	rs13387970	2:165304831	SCN2A	rs13012293	0.028
2:165268464	SCN2A	rs10197716	2:165270664	SCN2A	rs2119068	0.009
2:165268464	SCN2A	rs10197716	2:165290104	SCN2A	rs11889342	0.001
2:165290104	SCN2A	rs11889342	2:165330368	SCN2A	rs3769947	0.013
2:166053034	SCN1A	rs3812718	4:69096731	UGT2B7	rs12233719	0.017
2:166065518	SCN1A	rs13397210	4:69096731	UGT2B7	rs12233719	0.010
2:166071149	SCN1A	rs1020852	4:69096731	UGT2B7	rs12233719	0.013
2:165310162	SCN2A	rs2116658	4:69105219	UGT2B7	rs10028494	0.015
2:165337455	SCN2A	rs13387970	4:69107059	UGT2B7	rs5013211	0.026
2:165307150	SCN2A	rs7598931	4:69107059	UGT2B7	rs5013211	0.012
2:165307150	SCN2A	rs7598931	4:69096731	UGT2B7	rs12233719	0.001
2:165310162	SCN2A	rs2116658	4:69096731	UGT2B7	rs12233719	0.024

SNPs: single-nucleotide polymorphisms.

## Data Availability

The data used to support the findings of this study are available from the corresponding authors upon request.
